# Supplementary material for: trans-Translation inhibitors that kill Mycobacterium tuberculosis and pathogenic non-tuberculous mycobacteria also disrupt metal homeostasis
Source: Microbiology (Reading). 2026 May 21;172(5):001716. doi: 10.1099/mic.0.001716 (PMC13193620; doi:10.1099/mic.0.001716)
Supplement: Supplementary Material 1. [file mic-172-01716-s001.pdf]

trans-Translation inhibitors that kill _M. tuberculosis_ and pathogenic Non-tuberculous Mycobacteria also disrupt metal homeostasis

AUTHOR LIST:

Akanksha Varshney[1,‡], Ziyi Jia[2,‡], Gebremichal Gebretsadik[2], Narendran G-Dayanandan[3], Terry L. Bowlin[3], Michelle M. Butler[3], Anthony D. Baughn[2**], Kenneth C. Keiler[1,4*]

AUTHOR AFFILIATIONS AND FOOTNOTES:

[1] Department of Molecular Biosciences, The University of Texas at Austin, Austin, TX 78712 USA.

[2] Department of Microbiology and Immunology, University of Minnesota Medical School, Minneapolis, MN 55455 USA.

[3] Microbiotix, Inc., Worcester, MA 01605 USA

[4] LaMontagne Center for Infectious Diseases, University of Texas at Austin, Austin, TX

‡ These authors contributed equally

*Correspondence: kenneth.keiler@austin.utexas.edu

**Correspondence: abaughn@umn.edu

SUPPORTING INFORMATION

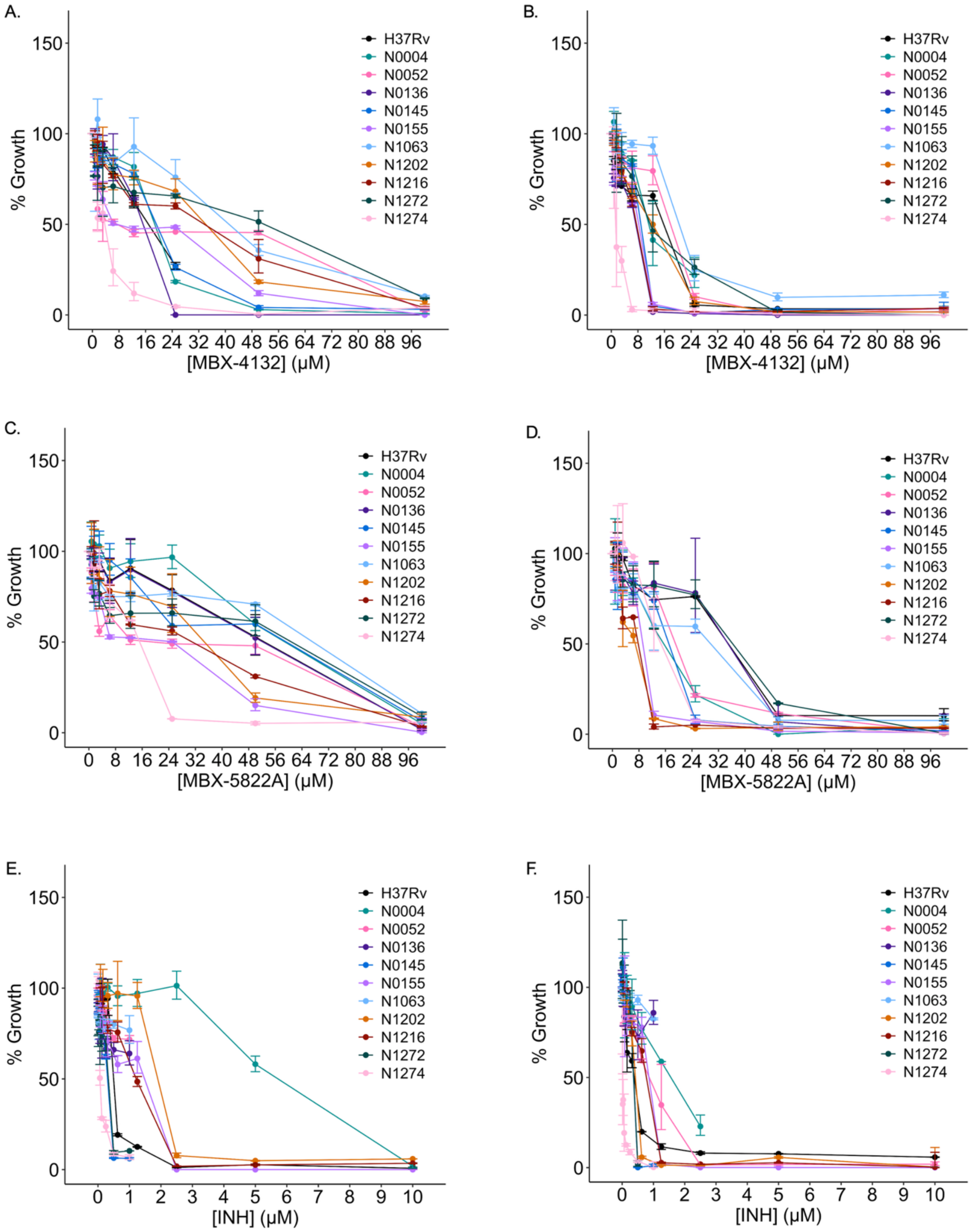

Figure S1. Acylaminooxadiazole inhibition against MTBC clinical isolates. Cells were treated with (A-B) MBX-4132, (C-D) MBX-5822A, or (E-F) INH for 7 days in (A,C,E) Middlebrook 7H9 or (B,D,F) 7H9 supplemented with 100 μM $ZnSO_4$. Data represent geometric means and geometric standard deviations for 2 biological replicates.

A.

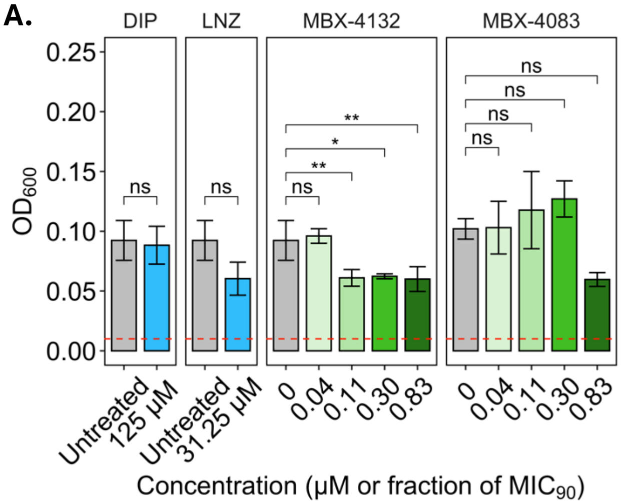

B.

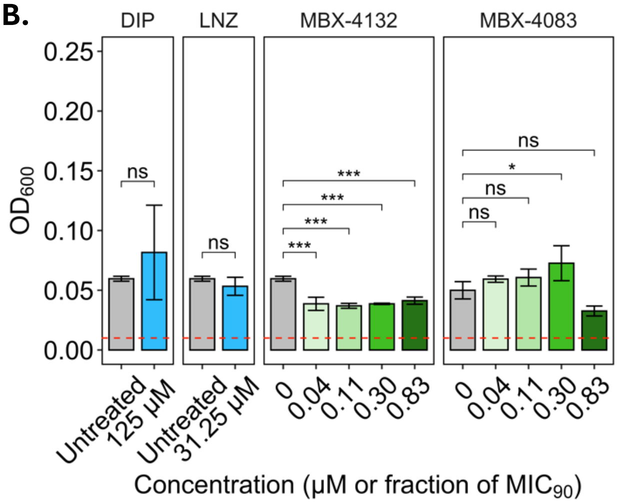

Figure S2. eGFP mutants did not experience strong growth impairment upon treatment with acylaminooxadiazoles. OD_{600} of (A) H37Rv *mbtH-egfp* and (B) H37Rv *whiB7-egfp* cultures upon treatment of DIP, LNZ, MBX-4132, or MBX-4083 was measured, as part of the fluorescent assays in Figure 7. Cells were treated in HZMM for 48 hours. Molar concentrations of DIP and LNZ were denoted, whereas MBX-4132 and MBX-4083 concentrations were shown as fractions of MIC_{90} in HZMM. Statistical analyses were done by comparing OD_{600} of drug-treated cultures to those of no-drug controls using Dunnett's Test. Data represent means and standard deviations of 3 biological replicates. Asterisks indicate statistical significance levels. ns $p \geq 0.05$, *$p < 0.05$, **$p < 0.01$, ***$p \leq 0.001$. Red dashed lines represent the starting OD_{600} of 0.01, prior to compound treatment.

SUPPLEMENTAL TABLES

Table S1. Minimum inhibitory and minimum bactericidal concentrations of *trans*-translation inhibitors for mycobacterial species in 7H9 medium

Compound	*M. tuberculosis* H37Rv Δ*RD1* Δ*panCD*		*M. avium*		*M. abscessus*	
	**MIC[a]**	**MBC[b]**	**MIC**	**MBC**	**MIC**	**MBC**
KKL-35	>40 (>125)	>40 (>125)	>40 (>125)	>40 (>125)	>40 (>125)	>40 (>125)
MBX-4132	>40 (>125)	>40 (>125)	>40 (>125)	>40 (>125)	>40 (>125)	>40 (>125)
MBX-5822A	40 (125)	40 (125)	40 (125)	40 (125)	>40 (>125)	>40 (>125)
MBX-6958A	40 (125)	40 (125)	40 (125)	>40 (>125)	>40 (>125)	>40 (>125)
MBX-6957	>40 (>125)	>40 (>125)	40 (125)	40 (125)	>40 (>125)	>40 (>125)
MBX-4370	>40 (>125)	>40 (>125)	40 (125)	40 (125)	40 (125)	40 (125)
MBX-4083	7.5 (25)	7.5 (25)	23 (75)	23 (75)	60 (200)	60 (200)
KKL-38989	32 (100)	32 (100)	>32 (>100)	>32 (>100)	>32 (>100)	>32 (>100)
KKL-39893	16 (50)	16 (50)	>32 (>100)	>32 (>100)	>32 (>100)	>32 (>100)
KKL-39894	32 (100)	32 (100)	>32 (>100)	>32 (>100)	>32 (>100)	>32 (>100)
KKL-39895	32 (100)	32 (100)	>32 (>100)	>32 (>100)	>32 (>100)	>32 (>100)
KKL-39896	32 (100)	32 (100)	>32 (>100)	>32 (>100)	>32 (>100)	>32 (>100)
Rifampicin	0.12 (0.14)	0.12 (0.14)	0.5 (0.6)	1 (1.2)	128 (155)	256 (311)
Azithromycin	4 (5.3)	ND[c]	32 (42.4)	ND	8 (10.6)	ND

[a]μg/mL (μM) values from at least three broth microdilution assays.

[b]μg/mL (μM) values from at least three plating assays.

[c]Not determined.

Table S2. Effect of Cu^{2+} on antibacterial activity of acylaminooxadiazoles in LIMM

Compound	*M. tuberculosis* H37Rv Δ*RD1* Δ*panCD*		*M. avium*		*M. abscessus*	
	**MIC[a]**	**MBC[b]**	**MIC**	**MBC**	**MIC**	**MBC**
KKL-35	>40 (>125)	>40 (>125)	>40 (>125)	>40 (>125)	>40 (>125)	>40 (>125)
MBX-4132	>40 (>125)	>40 (>125)	>40 (>125)	>40 (>125)	>40 (>125)	>40 (>125)
Rifampicin	0.12 (0.14)	0.12 (0.14)	0.5 (0.6)	1 (1.2)	128 (155)	256 (311)
Azithromycin	4 (5.3)	ND[c]	32 (42.4)	ND	8 (10.6)	ND

[a]μg/mL (μM) values from at least three broth microdilution assays.

[b]μg/mL (μM) values from at least three plating assays.

[c] Not determined.

Table S3. Effect of Cu^{2+} on antibacterial activity of acylaminooxadiazole in HZMM

Compound	MIC in MM[a]	MIC in HZMM[a]	MIC in HZMM + 1 μM $CuSO_4$[a]
MBX-4132[b]	>80 (>250)	1.3 (4)	>80 (>250)
MBX-5822A	>80 (>250)	<0.3 (<1)	>80 (>250)
MBX-6958A	>80 (>250)	<0.3 (<1)	>8 (>25)
MBX-4083	10 (31)	10 (31)	5 (16)

[a]μg/mL (μM) values from at least three broth microdilution assays against *M. tuberculosis* H37Rv.

[b]MBX-4132 MICs in MM and HZMM were described previously (25).

Table S4. Bacterial strains, plasmids and oligonucleotides

Strain	Description	Source or reference
M. tuberculosis N0004	Clinical isolate, lineage 3 from India	Gift from Berney Lab (Borrell *et al*., 2019; Glossop *et al*., 2025)
M. tuberculosis N0052	Clinical isolate, lineage 2.2.2 from China	Gift from Berney Lab (Borrell *et al*., 2019; Glossop *et al*., 2025)
M. tuberculosis N0136	Clinical isolate, lineage 4.3.3 from USA	Gift from Berney Lab (Borrell *et al*., 2019; Glossop *et al*., 2025)
M. tuberculosis N0145	Clinical isolate, lineage 2.2.1.1 from China	Gift from Berney Lab (Borrell *et al*., 2019; Glossop *et al*., 2025)
M. tuberculosis N0155	Clinical isolate, lineage 2.2.1 from China	Gift from Berney Lab (Borrell *et al*., 2019; Glossop *et al*., 2025)
M. africanum N1063	Clinical isolate, lineage 5, requires 40 mM pyruvate	Gift from Berney Lab (Guerrero-Bustamante *et al*., 2021; Glossop *et al*., 2025)
M. africanum N1202	Clinical isolate, lineage 6 from Ghana, requires 40 mM pyruvate	Gift from Berney Lab (Borrell *et al*., 2019)
M. tuberculosis N1216	Clinical isolate, lineage 4.6.2.2 from Ghana	Gift from Berney Lab (Borrell *et al*., 2019)
M. africanum N1272	Clinical isolate, lineage 5 from Ghana, requires 40 mM pyruvate	Gift from Berney Lab (Borrell *et al*., 2019; Glossop *et al*., 2025)
M. tuberculosis N1274	Clinical isolate, lineage 3 from Afghanistan, streptomycin resistant	Gift from Berney Lab (Borrell *et al*., 2019; Glossop *et al*., 2025)
M. tuberculosis Erdman	WT strain; virulent	Gift from Tischler Lab (Block *et al*., 2023)
M. tuberculosis Erdman *mbtF*::Tn	Erdman derivative; contains transposon insertion in *mbtF*	Gift from Tischler Lab (Block *et al*., 2023)
M. tuberculosis Erdman *mmpS5*::Tn	Erdman derivative; contains transposon insertion in *mmpS5*	Gift from Tischler Lab (Block *et al*., 2023)
M. tuberculosis Erdman *irtA*::Tn	Erdman derivative; contains transposon insertion in *irtA*	Gift from Tischler Lab (Block *et al*., 2023)
M. tuberculosis H37Rv *whiB7-egfp*	H37Rv derivative; pUMN105 integrated downstream of *whiB7*	This study
M. tuberculosis H37Rv *mbtH-egfp*	H37Rv derivative; pUMN105 integrated downstream of *mbtH*	This study
M. tuberculosis H37Ra	WT strain; avirulent	Gift from Jacobs Lab

M. tuberculosis H37Ra pKM461	H37Ra derivative; expresses RecT annealase and Bxb1 integrase; tetracycline-inducible	Previously constructed in Baughn Lab
E. coli HB101	For propagation of plasmids	(Glover, 1985)
E. coli HB101 pUMN105	Contains plasmid pUMN105	This study

**Plasmid**	**Description**	**Source or reference**
pUMN105	pKM468 derivative; expresses enhanced green fluorescent protein (eGFP); hyg^R	This study

**Oligonucleotide**	**Sequence 5' to 3'**	**Source or reference**
whiB7-egfp ORBIT targeting oligo	CGGGTTTCGCCGAGCCCGACGCGATCGTCGTCTGAGCCGGCTCGCGCCGGCGGGCGCACCATCGCGGGGGTTTGTACCGTACACCACTGAGACCGCGGTGGTTGACCAGACAAACCCATCCTCCTTCCTCCTTCTATGCAACAGCATCCTTGCGCGGACGTCCGCGCGGACGCTTGTGACTCACGATCGAGCCTTGGTCG	This study
mbtH-egfp ORBIT targeting oligo	GATTGTGCGTTTGGTTCAAACTACCGGTGCACGCGCCGGGCAAGTCTGTGTCGTAGCTGTGAGCGAGCGGGGTTTGTACCGTACACCACTGAGACCGCGGTGGTTGACCAGACAAACCCATCCTCCTTCCTCCTTTCAGTCCTCGACCATGGCGTCACGCAGGCTCTTCGGCCGCAGATCGGTCCAGTTCTTTTCCACGT	This study
